# The chloroplast genome sequence of *Magnolia kobus* DC. (Magnoliaceae)

**DOI:** 10.1080/23802359.2018.1450666

**Published:** 2018-03-14

**Authors:** Eunji Song, Suhyeon Park, Jongsun Park, Sangtae Kim

**Affiliations:** a Department of Biology, Sungshin University, Seoul, Korea;; b InfoBoss Co. Ltd, Seoul, Korea

**Keywords:** Chloroplast genome, *Magnolia kobus*, Magnoliaceae

## Abstract

As an endangered species, *Magnolia kobus* is distributed in Jeju island in Korea with only about 500–1000 individuals. In this study, we presented a complete chloroplast genome of *M. kobus* which is 159,443 bp and has four sub-regions: 87,484 bp of large single copy and 18,783 bp of small single copy regions are separated by 26,588 bp of inverted repeat regions including 113 genes (79 unique genes, four rRNAs and 30 tRNAs). Phylogenetic analysis using chloroplast genomes showed that *M. kobus* is a sister of *M. insignis* and *M. laevifolia* clade.

The Magnoliaceae is characterized by undifferentiated perianth (except sect. *Buergeria*), numerous stamens and carpels that are spirally arranged on an elongated receptacle (Nooteboom 1985). Recent classification system of Magnoliaceae (Figlar and Nooteboom 2004) suggests that there are two subfamilies (Liriodendroideae and Magnolioideae) in the family. According to the recent phylogenetic study, 11 distinctive major clades have been recognized with several basal polytomies in the subgenus Magnolioideae (Kim and Suh 2013).

*Magnolia kobus* DC. is a common and world-wide ornamental garden tree, however, its natural distribution is restricted in Japan and Korea. Especially, as endangered species, it is distributed in Jeju island in Korea with only about 500–1000 individuals. *M. kobus* is included in section *Buergeria,* in recent classification system (Figlar and Nooteboom 2004) and characterized by differentiated tepals.

We collected *M. kobus* at the natural population in Halla mountain in Jeju island N33°25′24.95”, E126°36′32.61”). Voucher specimen is deposited in Sungshin University Herbarium (SWU: *S. Kim 201137*). Total DNA was extracted from fresh leaves of *M. kobus* by using the Exgene^TM^ Plant SV mini kit (GeneAll, Seoul, Korea). Genome sequencing was performed using the GAIIx system (Solexa/Illumina, San Diego, CA). The NGS reads were matched and filtered against the chloroplast (cp) genome of *Liriodendron tulipifera* (Cai et al. 2006). The *de novo* assembled sequences were generated through three different assemblers, respectively: NGS Cell (Ver. 3.1.0; CLCBio, Denmark), ABySS (Ver. 1.2.5; Simpson et al. 2009), and Velvet (Ver. 1.0.15; Zerbino and Birney 2008). In a bid to utilize the advantages of three assembly programs, we aligned all contigs generated from three assemblers against the *L. tulipifera* cp genome using Sequencer 4.9 (Gene Code Corporation, Ann Arbor, MI). The completed cp genomes were annotated using DOGMA (Wyman et al. 2004) and were mapped with GenomeVx (Conant and Wolfe 2008). The *M. kobus* cp genome sequences were submitted to the GenBank (JX280396).

We generated around 24.5 Gbps sequences and obtained 122 Mbp short reads (ca. 760 X) as a filtered result. The cp genome of *M. kobus* is 159,443 bp and has four subregions: 87,484 bp of large single copy and 18,783 bp of small single copy regions are separated by 26,588 bp of inverted repeat regions. This cp genome includes 113 identified genes (79 unique genes, four rRNAs, and 30 tRNAs); 21 genes (six protein-coding genes, four rRNAs and 11 tRNAs) are duplicated in inverted repeat regions. The overall GC content is 39.28%. The phylogenetic tree was reconstructed with this cp genome and previously published cp genomes in Magnoliaceae (five in subgen. Magnolioideae and two in subgen. Liriodendroideae). Sequence alignment was conducted by MAFFT (Katoh and Standley 2013). The maximum likelihood (ML) analysis was performed using raxmlGUI version 1.3 (Figure 1; Silvestro and Michalak 2012). *Magnolia kobus* is a sister of a clade comprising *M. insignis* and *M. laevifolia*. The cp genome resource will provide important information on the studies of *M. kobus* in Jeju island, which is an endangered species in Korea and on the future phylogenomic studies in the family.

**Figure 1.**
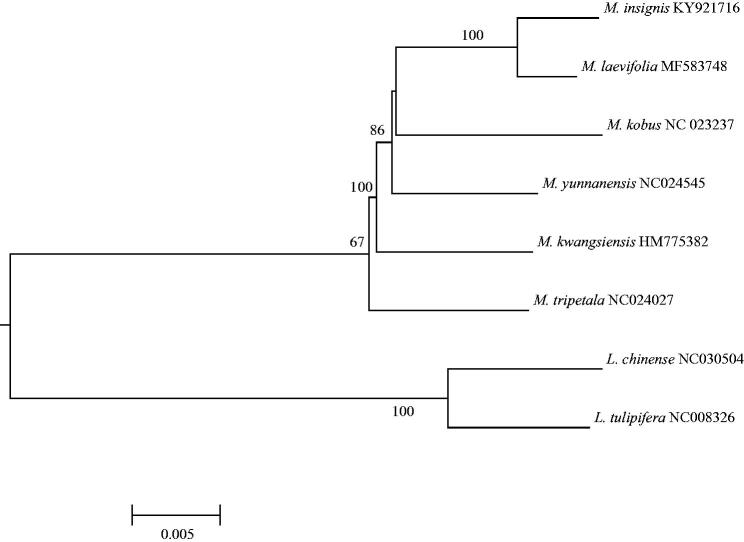
Maximum likelihood tree based on “GTR + gamma + I” model using seven previously published chloroplast genome sequences in Magnoliaceae (Cai et al. 2006; Kuang et al. 2011; Yang et al. 2014; Li et al. 2016; Zhu et al. 2016; Xu et al. 2017; Zheng and Xu 2017) and that of *M. kobus*. The tree is rooted by subfamily Liriodendroideae. The numbers above the node indicate bootstrap value (500 replicates; > 50% are indicated).
